# Hyaluronic Acid Profhilo^®^ Alleviates Skin Inflammation and Spinal Neuroimmune Alterations in a Mouse Model of Atopic Dermatitis

**DOI:** 10.3390/medicina62020405

**Published:** 2026-02-20

**Authors:** Gabriel Siquier-Dameto, Javier Gimeno-Beltrán, Gilberto Bellia, Andrea Giori, Pere Boadas-Vaello, Enrique Verdú

**Affiliations:** 1 Research Group of Clinical Anatomy, Embryology and Neuroscience (NEOMA), Department of Medical Sciences, University of Girona, 17003 Girona, Spain; info@dametoclinics.com; 2 Dameto Clinics International, 07460 Pollensa, Spain; 3 Pathology Department, Hospital del Mar, 08003 Barcelona, Spain; jgimenobeltran@psmar.cat; 4 IBSA Farmaceutici Italia, 26900 Lodi, Italy; gilberto.bellia@ibsa.it (G.B.); andrea.giori@ibsa.it (A.G.)

**Keywords:** atopic dermatitis model, hyaluronic acid, neuroimmune modulation, intradermal injection, calcitonin gene-related peptide (CGRP)

## Abstract

*Background and Objectives*: Hyaluronic acid (HA) is extensively used in dermo-aesthetic medicine for its hydrating and tissue-repairing properties. Beyond cosmetic use, HA has shown therapeutic effects in inflammatory skin diseases such as seborrheic, radiation-induced, and atopic dermatitis (AD). However, HA-based aesthetic formulations such as Profhilo^®^, a hybrid complex of high- and low-molecular weight HA, have not been tested in immunologically driven models of AD. This study aimed to investigate the therapeutic effects of intradermal Profhilo^®^ injections in a recently developed ovalbumin (OVA)-induced murine model of AD. Specific objectives included assessing changes in skin inflammation, pain sensitivity, and spinal cord pathology. *Materials and Methods*: Twenty-eight adult female ICR-CD1 mice were sensitized and exposed to OVA via intraperitoneal, subcutaneous, and topical routes over 49 days to induce AD-like lesions. Control animals received saline. On day 50, mice were subdivided into four groups receiving intradermal injections of Profhilo^®^ or saline. Skin inflammation was evaluated using the SCORAD index on days 49 and 57, and nociceptive responses were measured using the plantar thermal hyperalgesia test. On day 57, dorsal skin and thoracic spinal cord samples were collected for histological and immunohistochemical analysis, including assessments of epidermal and dermal thickness, mast cell density, collagen content, CGRP immunoreactivity, and microglial activation. *Results:* OVA-treated mice developed significant skin inflammation (*p* < 0.0001) and thermal hyperalgesia. Intradermal HA injection significantly reduced SCORAD scores (*p* < 0.01) and mast cell density (*p* < 0.05) while increasing dermal thickness (*p* < 0.05). In the spinal cord, HA treatment reduced CGRP immunoreactivity and microglial activation (*p* < 0.01 and *p* < 0.05, respectively), especially in OVA-treated animals. *Conclusions:* Intradermal Profhilo^®^ alleviated both cutaneous inflammation and neurogenic pain in an OVA-induced AD model. These findings suggest that HA not only improves local skin pathology but also modulates central neuroimmune responses, supporting its therapeutic potential for inflammatory skin conditions involving peripheral and central sensitization.

## 1. Introduction

Hyaluronic acid (HA) dermal injections are a common procedure in cosmetic dermatology to restore volume, reduce wrinkles, and improve facial contour, giving the skin a rejuvenated appearance [1,2]. This effect can be partly attributed to HA’s ability to attract and retain water, which helps to hydrate and plump the skin [2,3]. HA has also been used to treat certain skin conditions, such as dermatitis, including seborrheic dermatitis, where topical application of an HA cream or gel improved symptoms and signs (e.g., erythema and inflammatory skin lesions) [4,5]. In radiation dermatitis, HA gel also improved signs and symptoms [6,7], and in atopic dermatitis, transdermal application of HA via microneedle patches also improved symptoms [8,9]. In an experimental model of DNFB (2,4-dinitrofluorobenzene)-induced atopic dermatitis, HA creams of different molecular weights (2310 Da, 2697 Da, 7783 Da, 13253 Da, and 2 mDa) were tested. The best results, such as reduction in epidermal thickness and decrease in mast cells, were obtained with hyaluronic acids with molecular weights between 2310 and 7783 Da [10]. Similarly, in mice with atopic dermatitis, transdermal administration of HA (200–400 kDa) via microneedle patches reduced skin lesions [11]. Taken together, these studies provide strong evidence that HA application improves the signs and symptoms of various forms of dermatitis (seborrheic, atopic, and radiation-induced) by reducing skin inflammation.

In dermo-aesthetic and plastic surgery settings, various HA-based treatments are widely used, including Juvéderm^®^, Restylane^®^, Perlane^®^, and Profhilo^®^. Juvéderm™ (Allergan, Inc., Irvine, CA, USA), also known as Hydrafill, was approved by the FDA in 2006 for the correction of moderate to severe facial wrinkles and folds. It has been marketed in Europe and Canada since 2003. Juvéderm™ is derived from *Streptococcus equi* via bacterial fermentation and manufactured using “Hylacross technology,” Juvéderm is not subjected to a sizing process, where crosslinked HA is forced through a screen and broken into smaller fragments. It is cross-linked using 1,4-butanediol di-glycidyl ether (BDDE) [12]. Restylane^®^ (Medicis, Scottsdale, AZ, USA) was approved by the FDA in 2003 for similar indications, including nasolabial folds and cheek augmentation. Similar to Juvederm, it is derived via bacterial fermentation from *Streptococci* and uses BDDE for cross-linking [13]. Perlane^®^ (Medicis, Scottsdale, AZ, USA) is another BDDE-crosslinked HA product, derived from Streptococcus species. Approved by the FDA in 2007, it is used for implantation into the deep dermis and superficial subcutis to correct moderate to severe facial folds and wrinkles, such as nasolabial folds [12].

Clinical applications of these products include: (i) correction of nasolabial folds (Juvéderm, Restylane, Perlane^®^) [14,15,16,17,18,19]; (ii) lip volume enhancement (Juvéderm, Restylane) [20,21]; (iii) rejuvenation of skin on the hands and face (Juvéderm) [22,23,24]; (iv) restoration of malar volume (Juvéderm) [25], restoration of soft tissues and facial contour (Restylane) [26,27], and volume restoration in facial areas such as the periorbital, temporal, and glabellar regions (Perlane^®^) [28]; (v) treatment of facial lipoatrophy (Perlane^®^) [29]; (vi) cheek augmentation (Perlane^®^) [30]; (vii) correction of chin retrusion (Restylane) [31,32,33]; (viii) adjunct use in laryngoplasty (Juvéderm) [34] and vocal cord volume enhancement (Restylane) [35]; (ix) use as a scaffold in dental pulp regeneration (Restylane) [36]; and (x) nonsurgical rhinoplasty (Juvéderm) [37].

Profhilo^®^ is based on cooperative hybrid complexes of high- and low-molecular-weight HA, generated using NAHYCO technology. This technology enables the formation of stable cooperative hybrids by subjecting aqueous solutions containing high-molecular-weight (1100–1400 kDa) and low-molecular-weight (80–100 kDa) HA to a properly configured thermal cycle. The thermal cycle begins with heating the mixture to temperatures between 100 and 120 °C and holding it at that temperature for 10 min. After the thermal processing, the HA hybrid complexes remain stable over time and retain their rheological characteristics [38,39]. Clinical evidence shows that Profhilo^®^ has been used successfully to increase soft tissue volume in the facial region [40,41,42,43], to improve acne scars [44], to rejuvenate the skin of the neck and its appearance [45,46,47,48], to rejuvenate facial skin [48], and to improve the signs and symptoms of systemic sclerosis or scleroderma [49]. This product has also been injected into the clitoris [50] and into the vaginal walls [51] to address sexual dysfunction in women.

In atopic dermatitis (AD), a formulation combining hyaluronic acid (HA), soluble proteoglycan (900–1200 kDa) from salmon cartilage, and hydrolyzed collagen improved skin hydration, decreased the erythema index, and reduced pruritus [52]. Intradermal injection of Restylane^®^ in subjects with AD improved erythema and skin dryness [53]. In subjects with moderate AD, application of Hylatopic (Bausch Health US, LLC, Bridgewater, NJ, USA), an emollient foam based on hyaluronic acid, glycerin, and ceramides, significantly improved all signs of the disease compared to an emollient foam based on ceramide, cholesterol, and free fatty acids (Epiceram; Promius Pharma, Bridgewater, NJ, USA) [54]. In experimental models of atopic dermatitis (AD), such as that induced by 2,4-dinitrofluorobenzene (DNFB), treatment with a tacrolimus-conjugated hyaluronic acid (HA) cream improved skin hydration and reduced erythema, with less inflammatory cell infiltration and a reduction in epidermal hyperplasia and hyperkeratosis [55]. In this animal model, HA creams with molecular weights of 2310 Da, 2697 Da, and 7783 Da showed better results than those with higher molecular weights [10]. In atopic dermatitis (AD), a formulation combining hyaluronic acid (HA), soluble proteoglycan (900–1200 kDa) from salmon cartilage, and hydrolyzed collagen improved skin hydration, decreased the erythema index, and reduced pruritus [52]. Intradermal injection of Restylane^®^ in subjects with AD improved erythema and skin dryness [53], while in subjects with moderate AD, application of Hylatopic (Bausch Health US, LLC, Bridgewater, NJ, USA), an emollient foam based on hyaluronic acid, glycerin, and ceramides, significantly improved all signs of the disease compared to an emollient foam based on ceramide, cholesterol, and free fatty acids (Epiceram; Promius Pharma, Bridgewater, NJ, USA) [54]. In experimental models of atopic dermatitis (AD), such as that induced by 2,4-dinitrofluorobenzene (DNFB), treatment with a tacrolimus-conjugated hyaluronic acid (HA) cream improved skin hydration and reduced erythema, accompanied by less inflammatory cell infiltration and a reduction in epidermal hyperplasia and hyperkeratosis [55]. In this animal model, HA creams with molecular weights of 2310 Da, 2697 Da, and 7783 Da showed better results than those with higher molecular weights [10], which may be because HA > 50 kDa tends to hinder skin penetration. High molecular weight HA contributes to the formation of a film that prevents epidermal water loss, while low molecular weight HA penetrates better and helps restore a hydrated microenvironment for skin rejuvenation and tissue repair, possibly because HA above 50 kDa tends to hinder skin penetration. High molecular weight HA contributes to the formation of a film that prevents epidermal water loss, while low molecular weight HA penetrates better and helps restore a hydrated microenvironment for skin rejuvenation and tissue repair [56,57,58].

There are multiple animal models for AD, including genetically modified mice (e.g., IL4, IL31, caspase-1/IL18 transgenics, Apolipoprotein C1 transgenics, stratum corneum chymotryptic enzyme transgenics, thymic stromal lymphopoietin transgenics, KO-RelB, KO-Cathepsin E, KO-Filaggrin, and KO-Notch1/Notch2), animals that spontaneously generate AD (e.g., Nc/Nga mouse, Naruto Research Institute Otsuka (NAO) mice, Flaky Tail (ft/ft) mice with mutations in the filaggrin (flg) and matted (ma) genes, and DS-Nh mice); animal models in which AD is induced by epicutaneous sensitization using various substances (e.g., oxazolone, trinitrochlorobenzene, 2,4-dinitroflourobenzene, fluorescein isothiocyanate, MC903 or calcipotriol, and ovalbumin) or house dust mites (HDM; e.g., *Dermatophagoides farina* are acariform mites belonging to the Pyroglyphidae family); and animal models where AD is induced by diet (e.g., 12-O-tetradecanoylphorbol-13-acetate (TPA), squaric acid dibytylester (SADBE), and urushiol) [59,60,61,62,63,64].

In ovalbumin-induced AD models in mice and rats, sensitization protocols generally involve: (i) intraperitoneal injections of ovalbumin combined with patches (1 × 1 cm or 1.5 × 1.5 cm) impregnated with ovalbumin solution and attached to shaved and depilated dorsal skin [65,66,67,68,69,70]; (ii) intraperitoneal sensitization with ovalbumin, followed by subcutaneous injection of ovalbumin into the dorsal skin and application of patches [71]; and (iii) repeated intraperitoneal injections of ovalbumin, followed by subcutaneous injections of ovalbumin and skin impregnation with ovalbumin using a brush on shaved and depilated dorsal skin [72].

Currently, HA compounds used in dermo-aesthetics (e.g., Juvéderm, Restylane, Perlane, and Profhilo) have not been studied in ovalbumin-induced atopic dermatitis models. The primary objective of this study is to evaluate the effects of intradermal Profhilo injections in the recently reported experimental model of ovalbumin-induced atopic dermatitis [72]. The specific objectives of this study are: (i) to determine dorsal skin alterations using the SCORAD scale in ovalbumin-treated animals and to analyze Profhilo’s effect; (ii) to assess changes in epidermal and dermal thickness, mast cell density, and collagen fiber area (Masson’s trichrome) after intradermal Profhilo injection; (iii) to study whether repeated treatment with ovalbumin solution—administered through intraperitoneal injection, subcutaneous injection, and topical application using a brush on the dorsal skin—induces pain-related responses such as plantar thermal hyperalgesia; and (iv) to explore whether dorsal skin changes influence the spinal cord (e.g., gliosis and CGRP-positive fiber sprouting in the dorsal horn) and whether Profhilo injections modulate these changes. The main novelty of this study is the use of intradermal Profhilo injections in ICR-CD1 mice with ovalbumin-induced atopic dermatitis.

## 2. Materials and Methods

### 2.1. Animals, Experimental Design, and Ethical Regulations

In this study, adult female ICR-CD1 mice (8 weeks old, weighing 25–30 g) obtained from Janvier Laboratories (Le-Genest-Saint Isle, France) were used. A total of 28 mice were included: 14 mice were treated with saline solution, and 14 mice with ovalbumin solution, following the procedure described above [72]. Briefly, on days 0, 7, and 14, animals received 0.2 mL of ovalbumin solution with aluminum salts intraperitoneally. Control animals received an equivalent volume of saline solution. On day 14, after anesthesia with sodium pentobarbital (# Y0002194, Sigma-Aldrich-Merck, Darmstadt, Germany; 50 mg/kg; 10 mg/mL; i.p.), the dorsal skin was shaved and depilated, using depilatory cream (Silky fresh sensitive skin; Veet, Reckitt Benckiser Healthcare, Hull, UK). The dorsal skin was then impregnated with an ovalbumin solution and aluminum salts using a Pelican brush (nº. 10; Pelikan 721431; Hannover, Germany). On days 15–21, 28–35, and 42–49, the ovalbumin-treated animals received daily 0.2 mL of ovalbumin solution intraperitoneally, 0.2 mL of ovalbumin solution with aluminum salts subcutaneously, and topical application of the same ovalbumin solution (with aluminum salts) to the dorsal skin using a brush. Control animals were treated in the same way but received saline solution only.

Plantar thermal hyperalgesia was assessed on days 21, 35, and 49. On day 50, after anesthesia with sodium pentobarbital, the dorsal skin of the animals was shaved and depilated again, and the degree of skin damage was evaluated using the SCORAD score method adapted for rodents. After this evaluation, the 14 ovalbumin-treated mice were divided into two subgroups: (i) one group received intradermal injections of hyaluronic acid (Profhilo^®^, IBSA; OVA + HA group, n = 7), and (ii) the other group received intradermal injections of sterile saline (OVA + Saline group, n = 7). The saline-treated mice were also divided into two subgroups: (i) one group received intradermal hyaluronic acid (Saline + HA group, n = 8); and (ii) the other received sterile saline (Saline + Saline group, n = 6).

On day 57, plantar thermal hyperalgesia and skin damage (SCORAD) were assessed again. Then under deep anesthesia (sodium pentobarbital (90–100 mg/kg; 10 mg/mL; i.p.)), the mice were perfused intraventricularly with paraformaldehyde in phosphate-buffered saline. The dorsal skin and spinal cord were extracted and processed for histological analysis.

All in vivo experimental procedures were performed at the Bellvitge animal facility of the University of Barcelona. The protocol was approved by the Animal Experimentation Ethics Committee of the University of Barcelona (CEEA; CEEA number 50/19; approved on 11 April 2019) and by the Department of Agriculture, Livestock, Fisheries, Food, and the Natural Environment of the Catalan government (DAAM number 10672; approved on 22 November 2019). The study complies with the ARRIVE 2.0 guidelines, the ethical principles of the International Association for the Study of Pain (IASP) for the use of animals in pain research [73], and the Directive (2010/63/EU) of the European Parliament and Council.

Regarding the housing conditions of the animals used in this study, the mice were housed in two types of Panlab cages: the Panlab type 500 mouse cage (polycarbonate; Ref: PB101202; Panlab, Barcelona, Spain), with internal dimensions of 220 × 220 × 145 mm (height) and a base surface area of 500 mm^2^, covered with a stainless steel grid (Ref: PB101509; Panlab, Barcelona, Spain), and a food and drink separator, also made of stainless steel (Ref: PB101614; Panlab, Barcelona, Spain), and the Panlab type 1000 mouse cage (Ref: PB101205; Panlab, Barcelona, Spain), made of polycarbonate, with internal dimensions of 215 × 465 × 145 mm (height) and a base surface area of 1000 mm^2^, covered with a stainless steel grid (Ref: PB101510; Panlab, Barcelona, Spain), and a food and drink separator, also made of stainless steel (Ref: PB101614; Panlab, Barcelona, Spain). Only 3 mice were placed in the 500-type cage, while a maximum of 5 mice were placed in the 1000-type cage. A bedding of spruce wood shavings with a sieve size > 0.71 mm and an absorption rate of 97% was used (Safe-Prime S; Safe-Lab, Rosenberg, Germany). The diet consisted of maintenance pellets measuring 16.4 mm in diameter and 22.6 mm in length, with an average weight of 5.3 g (Safe-A04; Safe-Lab, Rosenberg, Germany). Water was supplied via a 500 mL polycarbonate bottle with a stainless-steel nozzle. The water used was tap water purified through special filters, which circulated in a different distribution system than unpurified tap water. The animal housing technicians changed the cages and bedding once a week, and food and water were provided ad libitum. Each day, this technical staff inspected all the cages and addressed any issues. The animal housing area had a temperature of 21 ± 1 °C, with an air humidity of 40–60%, forced air renewal, and a 12:12 light: dark cycle (starting at 8:00 a.m. and ending at 8:00 p.m.), that is, 8 a.m. to 8 p.m. light and 8 p.m. to 8 a.m. darkness. The transition from one situation to the other was gradual, not abrupt. Finally, nesting materials (paper tissues) and cellulose sticks for gnawing, provided by the technical service, were used as enrichment resources.

When anesthesia has been required, it has always been by using intraperitoneal injection of sodium pentobarbital (50 mg/kg; 10 mg/mL), while euthanasia of the animals has been carried out by overdose of sodium pentobarbital (200 mg/kg; 40 mg/mL) and subsequent cervical luxation (all these anesthesia and euthanasia procedures are according to Royal Decree (RD) 53/2013 on the basic rules applicable for the protection of animals used in experimentation and other scientific purposes, including teaching, of the Government of Spain).

On the other hand, the animal monitoring protocol used contains the following sections: (i) body weight loss [Normal or no weight loss = 0 points; loss < 10% = 1 point; loss 10–20% = 2 points; loss > 20% = 9 points]; (ii) animal appearance [Normal = 0 points; skin or hair in poor condition = 1 point; skin or hair in poor condition and presence of nasal and/or ocular secretions = 2 points; abnormal posture = 9 points]; (iii) unprovoked behavior [Normal = 0 points; animal that grooms little or that has bristled hair or hunched posture with closed or half-closed eyes = 1 point; inactivity = 2 points; self-mutilation, abnormal vocalization, or very restless or immobile animal = 9 points]; and (iv) Behavior in response to handling [Normal = 0 points; vocalization during handling = 1 point; agitated, tense, nervous animal with vocalization during handling = 2 points; aggressive or comatose animal with vocalization during handling = 9 points]. The final score for each animal is the sum of the scores obtained in each of the four sections above. Any animal with a score of 9 points or higher during the experimental days was removed from the procedure and euthanized. Several interventions were also considered based on a range of scores in this monitoring protocol: (i) no intervention (0–4 points); (ii) the animal is monitored twice daily and administered glucose saline solution (0.5 mL; i.p.) (5–8 points); (iii) severe suffering and euthanasia (9 points or higher). This monitoring protocol and its corrective measures were applied from the first time ovalbumin (OVA) was applied cutaneously, and the frequency was daily during the OVA application period and until the end of the functional follow-up. The researchers themselves were responsible for implementing this monitoring protocol.

### 2.2. Preparation of Ovalbumin Solutions and Treatment with Hyaluronic Acid

For the ovalbumin solution (OVA solution), 0.5 g of ovalbumin (grade V; #A5503; Sigma-Aldrich-Merck, Darmstadt, Germany) was dissolved in 500 mL of sterile saline (0.9% Vitulia Physiological Serum, Barcelona, Spain). For the ovalbumin aluminum salt solution (OVA-AL solution), 0.5 g of ovalbumin and 0.2 g of aluminum hydroxide hydrate (#A1577; Sigma-Aldrich-Merck, Darmstadt, Germany) were dissolved in 500 mL of sterile saline.

All solutions were filtered using 0.22 μm syringe filters (#SFNY-122-100; Labbox, Barcelona, Spain), aliquoted into 50 mL tubes (#PTSP-E50-025; Labbox, Barcelona, Spain), and stored at 4 °C until use.

Regarding treatment with HA, Profhilo^®^ (Institut Biochimique SA, IBSA, Via della Filanda, 30 26900 Lodi, Italy) was administered intradermally under aseptic conditions. A sterile 1 mL syringe was loaded with HA, and a 34 G invisible needle (TSK, Gemullehoekenweg 42 Oisterwijk, Noord-Brabant 5062CD, The Netherlands) was affixed. Each animal received 0.05 mL of HA at four dorsal injection sites (total of 0.2 mL per animal) on day 50, under anesthesia, with the dorsal skin previously shaved and depilated. Control animals received 0.2 mL of sterile saline intradermally in the same manner.

Up to day 49, there were two experimental groups: (i) mice treated cutaneously with ovalbumin solution and (ii) mice treated with saline solution. From day 50 onward, four final experimental groups were established: (i) Saline + HA (cutaneous treatment with saline solution and intradermal injection with HA), (ii) Saline + Saline (cutaneous treatment with saline solution and intradermal injection with saline), (iii) OVA + HA (cutaneous treatment with OVA and intradermal injection with HA), and (iv) OVA + Saline (cutaneous treatment with OVA and intradermal injection with saline).

### 2.3. Evaluation of Plantar Thermal Hyperalgesia and Evaluation of Cutaneous Alterations

Plantar thermal hyperalgesia was assessed on days 21, 35, 49, and 57 of follow-up using a plantar test device (#37370; Ugo Basile, Gemonio, Italy). The final measurement (day 57) was performed one week after the intradermal injection of HA or saline solution.

For the assessment, mice were placed in glass-bottomed methacrylate boxes and allowed to acclimatize for 60 min. A beam of incandescent light from a 100 W bulb was then directed at the plantar surface of the hind paws, and the withdrawal latency to this painful stimulus was determined. To prevent burns, exposure time was limited to a maximum of 30 s. The average withdrawal latency for both hind paws was calculated from three independent trials, each performed at 5-min intervals. In this test, a shorter withdrawal latency indicates a higher degree of thermal hyperalgesia [74,75].

The degree of skin alteration was assessed using the SCORAD score (SCORing Atopic Dermatitis) adapted for rodents. The SCORAD system evaluates the severity of erythema/hemorrhage, scarring/dryness, edema, and excoriation/erosion on the dorsal skin. Each parameter is scored as 0 (none), 1 (mild), 2 (moderate), or 3 (severe). The total score, ranging from 0 (no dermatitis) to 12 (very severe dermatitis), reflects the overall severity of skin inflammation [72,76]. As described above, SCORAD evaluations were performed on day 49 and again on day 57, one week after the intradermal injection of hyaluronic acid or saline.

### 2.4. Histological Evaluation of Dorsal Skin and Spinal Cord

On day 57 of follow-up, one week after the intradermal injection of HA or saline solution and following functional and dermatological assessments, animals were deeply anesthetized with sodium pentobarbital and intraventricularly perfused with 4% paraformaldehyde (# P6148, Sigma-Aldrich-Merck, Darmstadt, Germany) in phosphate-buffered saline (PBS, 0.1 M, pH = 7.4).

The dorsal skin was carefully removed and placed in a glass vial filled with the same fixative, then stored at 4 °C for 25 days. The spinal cord was extracted via dorsal laminectomy, and the thoracic segment was isolated using the ribs as anatomical landmarks. These spinal cord segments were also placed in glass tubes with the same fixative and stored at 4 °C for 25 days. After fixation, both tissues were transferred to fresh containers filled with a cryoprotectant solution (30% sucrose in PBS, 0.1 M, pH = 7.4) and kept at 4 °C for 25–30 days.

The dorsal skin was then stretched over cork and pinned at the edges to ensure maximal tension. A 2 cm^2^ central section was excised and embedded in Tissue Freezing Medium (# 0201-08-926; Leica, Barcelona, Spain). Blocks were frozen in a cryostat (CM1520, Leica, Barcelona, Spain) at −24 °C. Skin was cryo-sectioned at 20 μm thickness, and 8–10 sections per animal were mounted on pregelatinized slides. Sets of histological sections were processed using conventional histological staining techniques.

Spinal cord segments (2–3 mm; thoracic region) were also embedded in Tissue Freezing Medium, frozen at −24 °C, and sectioned at 60 μm using a cryostat (CM1520, Leica, Barcelona, Spain). These sections were collected in 6-well porcelain plates (#SPPC-006-001; Labbox, Barcelona) and processed using immunohistochemical methods.

#### 2.4.1. Processing of Dorsal Skin

One set of histological sections was stained with hematoxylin and eosin (H&E). Sections were first stained with hematoxylin (#HEMA-HPS-500; Labbox, Barcelona, Spain) for 30 s, followed by rinsing with running tap water to remove excess stain. After a 5-min bath in 70% ethanol, the sections were stained with eosin (#EOYDE-S0D-500; Labbox, Barcelona, Spain) for 2 min. Finally, the stained sections were dehydrated through a graded ethanol series (70°, 96°, 100°, 100°; 5 min per bath), cleared in xylene for 2 min, and coverslipped using DPX mounting medium (#1.01.979.500; Merck, Darmstadt, Germany).

Another set of dorsal skin sections was stained with commercial Giemsa solution (#EOMB-MSD-1K0; Labbox, Barcelona, Spain) diluted in distilled water (1:4 Giemsa:water) for 90 min. Sections were then rinsed with 0.1% acetic acid and 96% ethanol for 15–20 s each. This was followed by three 2 min methanol baths and three 2 min xylene baths. The coverslips were mounted with DPX.

A third set of histological sections was stained using Masson’s trichrome (#TRIC kmA-100, Labbox, Barcelona, Spain), following the manufacturer’s protocol. After staining, the sections were rinsed with distilled water to remove excess dye and dehydrated in graded ethanol (5 min per bath). Finally, they were cleared in xylene for two minutes, and coverslips were mounted with DPX.

Histological images of all stained sections were acquired using an optical microscope (Leica DMR-XA; Leica Microsystems, Barcelona, Spain) equipped with a digital camera (FMVU-13S2CCS, Point Gray Research, Canada). Images of histological sections stained with hematoxylin and eosin were used to measure epidermal and dermal thickness, following the method described by Siquier-Dameto et al. [72]. Giemsa-stained sections were used to count mast cells in the dermis; results were expressed as mast cell density (# mast cells/mm^2^). In addition, Masson’s trichrome-stained sections were analyzed to determine the stained area within the epidermis and dermis, which contains collagen fibers (types I and IV) deposited in these parts of the skin [77,78]. All images were processed using ImageJ (version 2.14.0/1.54f), a free image analysis software.

#### 2.4.2. Spinal Cord Processing

Histological sections of spinal cord were collected in 6-well porcelain dishes. After washing at room temperature with shaking, the sections were sequentially incubated in PBS (10 min), PBS + 0.3% Triton-X-100 (10 min), and PBS-Triton 479 + 1% fetal bovine serum (30–45 min). They were then incubated with primary antibodies: rabbit anti-ionized calcium-binding adapter molecule type 1 (Iba1; 1:200; #019-19741; WAKO, Richmond, VA, USA) to visualize microglia cells and goat anti-calcitonin gene-related protein (CGRP; 1:200; #ab36001, ABCAM, Cambridge, UK) to visualize peptidergic nociceptive afferent fibers in the dorsal horn of the spinal cord. Incubation with primary antibodies was performed for 48 h at 4 °C in a humid chamber with constant agitation.

Following three 10 min washes in PBS containing 0.3% Triton-X-100, the histological sections were incubated for 24 h at 4 °C in a humidified chamber with constant agitation with secondary antibodies: donkey anti-goat and donkey anti-rabbit conjugated with cyanine 3.18 (Cy3; 1:200; Jackson Immunoresearch, West Grove, PA, USA). After several additional washes with PBS-Triton and PBS, the sections were mounted on pre-gelatinized slides, dehydrated through graded ethanol (70°, 96°, then absolute), and coverslipped with DPX [75].

Spinal cord sections were examined under a Leica DMRXA epifluorescence microscope equipped with a digital camera (FMVU-13S2C-CS). Using ImageJ software (NIH, Bethesda, MD, USA; version 2.14.0/1.54f), the CGRP immunoreactive area in the dorsal horn of the spinal cord was measured, and reactive versus non-reactive microglia cells were counted to determine the percentage of reactive microglia as an indicator of microgliosis [75]. Microglia cells exhibiting branched or hyperbranched morphologies with fine processes around the soma were classified as non-reactive microglial cells, whereas amoeboid or sparsely branched microglial cells were considered reactive microglial cells [79,80].

### 2.5. Statistical Analysis

Functional and histological analyses were performed blindly, using a numerical code for each animal. Statistical comparisons between experimental groups were made using the Kruskal–Wallis and Mann–Whitney U non-parametric statistical tests. Results are shown as mean ± standard error of the mean (SEM), with statistical significance set at *p* < 0.05. Analyses were performed using GraphPad Prism 10 (version 10.5.0) for Macintosh.

## 3. Results

### 3.1. Cutaneous Alterations Assessed Using the SCORAD Score

During the 49-day follow-up period, saline-treated animals showed no signs of skin alterations, and their SCORAD score remained at 0 on days 21, 35, and 49. In contrast, ovalbumin-treated animals exhibited progressive skin changes.

**Figure 1:**
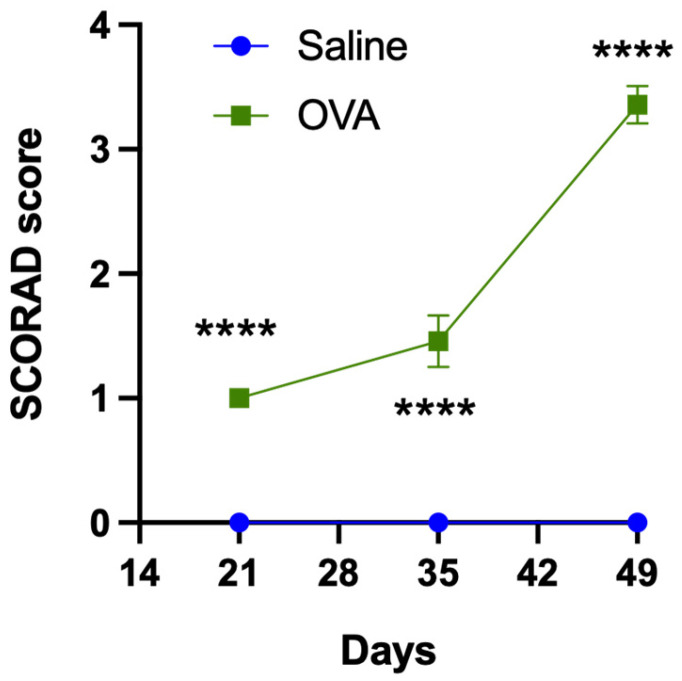
Skin alterations assessed using the SCORAD score in animals treated with saline or ovalbumin. In animals treated with saline solution, no skin alterations were observed on the SCORAD score scale; therefore, the score on the different days of follow-up was 0 points. In contrast, animals treated with ovalbumin (OVA) showed skin alterations on the SCORAD score scale, which increased over the days of follow-up. Significant differences were observed between the two experimental groups on the different days of follow-up. Values are expressed as mean ± standard error of the mean (SEM). Number of animals in each experimental group: 14 (n = 14). **** *p* < 0.0001 compared to the control group (saline).

On day 21, animals displayed either mild erythema or dry skin, resulting in a SCORAD score of 1 point. By day 35, the number of animals with erythema and dry skin had increased, raising the average SCORAD score to 1.46. On day 49, erythema progressed to a moderate level, mild dry skin persisted, and some animals also developed mild edema. The SCORAD score reached 3.36. Compared to saline-treated animals, the SCORAD scores in ovalbumin-treated animals were significantly higher (*p* < 0.0001) (Figure 1).

**Figure 2:**
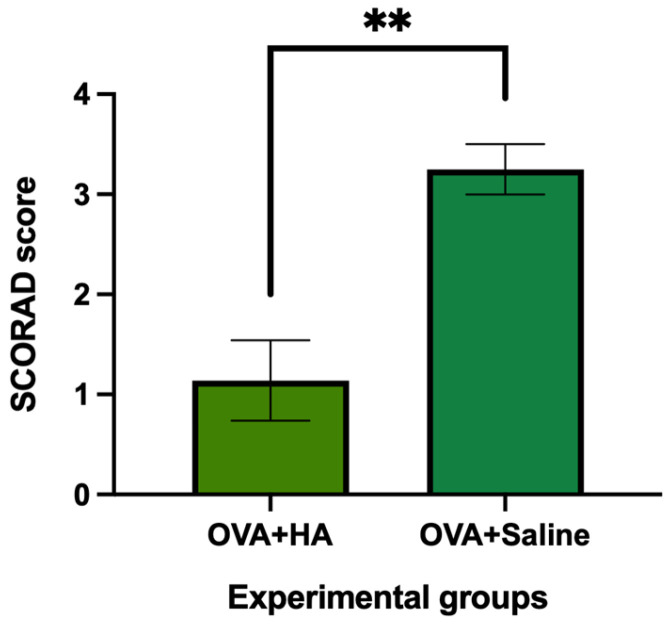
SCORAD score in ovalbumin-treated animals after intradermal injection of HA (OVA + HA group, n = 7) or saline solution (OVA + saline group, n = 7). The histograms show the mean SCORAD score and their respective mean errors in ovalbumin (OVA)-treated animals that received either hyaluronic acid (HA) or saline injection at 57 days of follow-up. Hyaluronic acid injection significantly reduced the SCORAD score compared to animals that received saline injection. Values are expressed as mean ± standard error of the mean (SEM). ** *p* < 0.0001 compared to the OVA + saline group.

On day 57, following intradermal injection of HA or saline, animals that previously received cutaneous treatment with saline showed no signs of skin alterations, and their SCORAD scores remained at 0. In contrast, among the animals previously treated with ovalbumin, those that received an intradermal injection of HA significantly reduced their SCORAD score compared to those that received an intradermal saline injection (*p* < 0.01) (Figure 2).

These results suggest that cutaneous application of ovalbumin induces significant skin alterations in the dorsal region compared to saline treatment. Furthermore, intradermal injection of HA into dorsal skin significantly alleviates these alterations, as reflected by the SCORAD score.

### 3.2. Histological Changes in the Dorsal Skin Following Cutaneous Treatment with Saline or Ovalbumin and Intradermal Injection of HA or Saline

**Figure 3:**
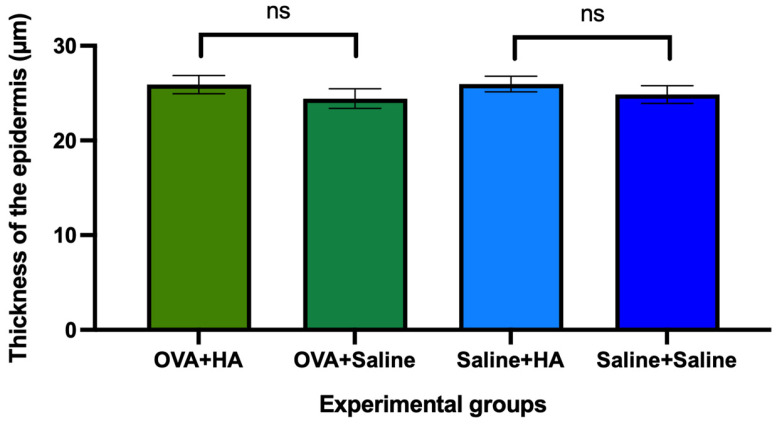
Epidermal thickness of dorsal skin in the different experimental groups on day 57 of follow-up. The histograms show the mean epidermal thickness (µm) and their respective mean errors in animals treated with ovalbumin (OVA) or saline solution and those that received a cutaneous injection of hyaluronic acid (HA) or saline solution. No significant differences (ns) were observed between animals injected with HA or saline, or between animals treated with OVA or those treated with saline. Values are expressed as mean ± standard error of the mean (SEM). Group sizes: OVA + HA (n = 7), OVA + saline (n = 7), saline + HA (n = 8), and saline + saline (n = 6); ns = not significant. No significant differences in dorsal skin epidermal thickness were observed between any of the experimental groups on day 57.

Histological analysis of epidermal thickness revealed no significant differences between saline-treated animals that received intradermal injections of either HA or saline. Similarly, in animals previously treated cutaneously with ovalbumin, epidermal thickness did not differ between those that received intradermal injections of HA and those that received saline (Figure 3).

**Figure 4:**
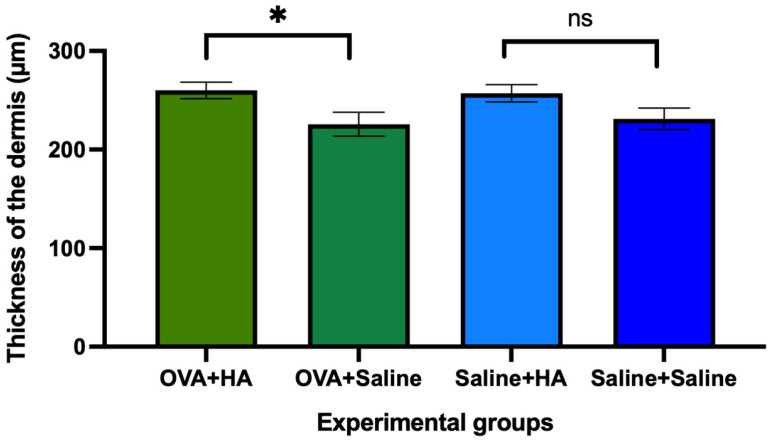
Dermal thickness of dorsal skin in the different experimental groups on day 57 of follow-up. The histograms show the mean dermal thickness (µm) and their respective mean errors in animals treated with ovalbumin (OVA) or saline solution that received a cutaneous injection of hyaluronic acid (HA) or saline solution. In animals treated with OVA, differences were observed between those that received HA injection and those that received saline, while no differences (ns) were observed between those that received HA injection and those that received saline. Values are expressed as mean ± SEM. Group sizes: OVA + HA (n = 7), OVA + saline (n = 7), saline + HA (n = 8), and saline + saline (n = 6); ns = not significant. *p* < 0.05 compared to the OVA + saline group. * *p* < 0.05.

The thickness of the dermis in the dorsal skin showed a significant difference between the OVA + saline and OVA + HA groups (*p* < 0.05), but not between the saline + saline and saline + HA groups (*p* > 0.05) (Figure 4). These results suggest that in animals previously treated cutaneously with ovalbumin, intradermal injection of HA significantly increases dermal thickness. A similar increase was observed in saline-treated animals injected intradermally with hyaluronic acid, although this difference was not statistically significant compared to the saline + saline group.

**Figure 5:**
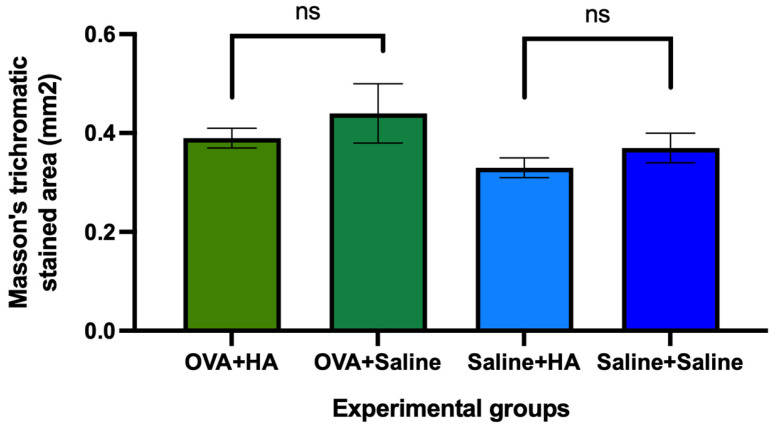
Area of dorsal skin (mm^2^) stained with Masson’s trichrome in the different experimental groups on day 57 of follow-up. The histograms show the mean skin area (mm^2^) stained with Masson’s trichrome, which stains collagen fiber deposits, and their respective mean errors in animals treated with ovalbumin (OVA) or saline solution and those that received cutaneous injection of hyaluronic acid (HA) or saline solution. No significant differences (ns) were observed between animals injected with HA or saline solution in mice treated with OVA and those treated with saline solution. Values are expressed as mean ± SEM. Group sizes: OVA + HA (n = 7), OVA + saline (n = 7), saline + HA (n = 8), and saline + saline (n = 6); ns = not significant.

Masson’s trichrome staining was used to assess collagen fiber deposition in the dermis and epidermis. In the dorsal skin of animals that received intradermal injection of HA, a trend toward decreased staining intensity was observed compared to animals injected with saline. However, no significant differences were observed between the experimental groups (Figure 5).

**Figure 6:**
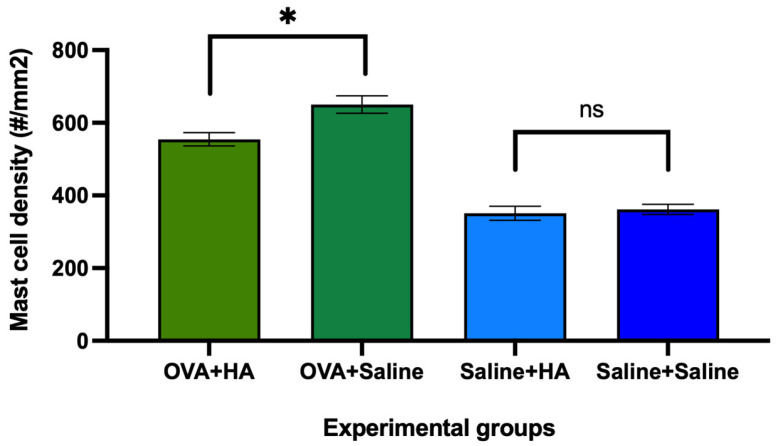
Density of mast cells (#/mm^2^) in the dorsal skin of animals on day 57 of follow-up. The histograms show the mean mast cell density (#/mm^2^) and their respective mean errors in the dorsal skin of mice treated with ovalbumin (OVA) or saline solution, which in turn received hyaluronic acid (HA) injection or saline solution. Differences were observed between the animals treated with OVA and those that received HA injection or saline solution, while no significant differences (ns) were observed between the animals treated with saline solution and those that received HA injection or saline solution. Values are expressed as mean ± SEM. Group sizes: OVA + HA (n = 7), OVA + saline (n = 7), saline + HA (n = 8), and saline + saline (n = 6). *p* < 0.05 compared to the OVA + saline group; ns = not significant. * *p* < 0.05.

Mast cell density in the dorsal skin was also assessed. In animals previously treated cutaneously with ovalbumin, intradermal injection of HA significantly reduced the density of mast cells compared to the OVA + saline group (*p* < 0.05). A similar trend was observed in saline-treated animals that received intradermal HA, but the difference was not statistically significant (Figure 6).

Furthermore, a significant increase in mast cell density was observed in animals treated cutaneously with ovalbumin compared to those treated cutaneously with saline (*p* < 0.0001), regardless of whether they received intradermal injections of HA or saline. In other words, significant differences were observed between the OVA + HA and saline + HA groups and between the OVA + saline and saline + saline groups.

**Figure 7:**
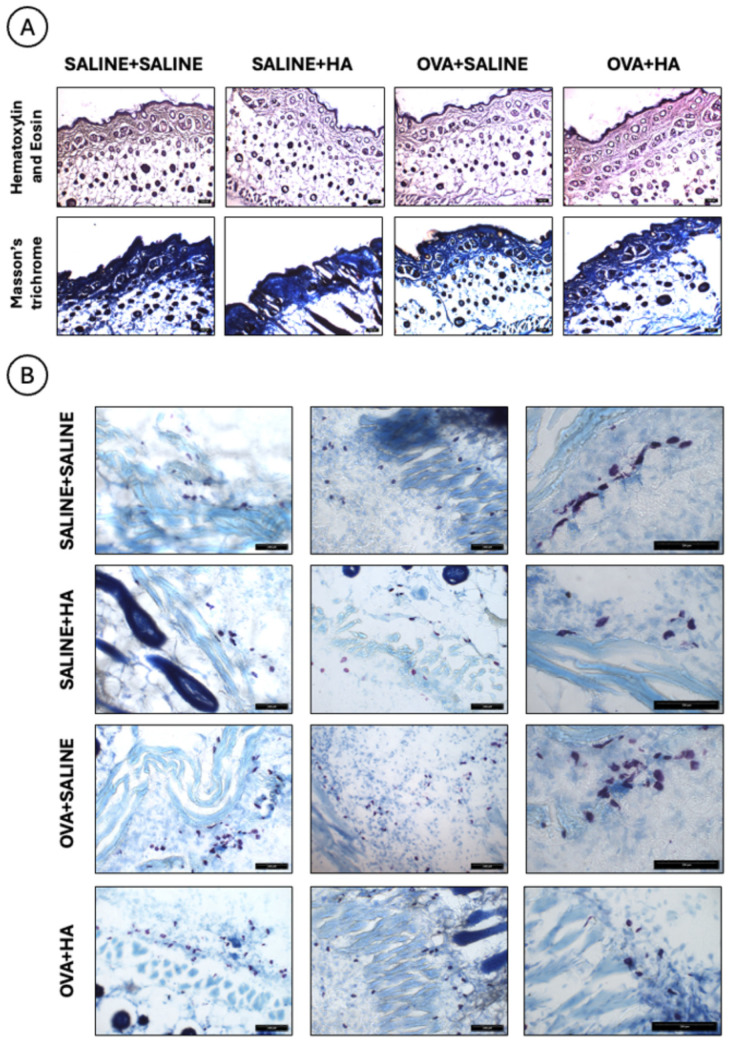
Histological images of dorsal skin after intradermal injection of HA or saline in animals treated with ovalbumin or saline. ((**A**), **top**) Sections stained with hematoxylin and eosin show the epidermis as a deep purple superficial band, with the dermis underneath. ((**A**), **bottom**) Sections stained with Masson’s trichrome highlight collagen in blue, mainly within the epidermis and dermis. (**B**) Giemsa-stained sections show mast cells in violet (left and center columns), with degranulating mast cells present in all groups (right column). Group sizes: OVA + HA (n = 7), OVA + saline (n = 7), saline + HA (n = 8), and saline + saline (n = 6). Scale bars = 100 µm.

Figure 7 shows histological images of dorsal skin sections from the different experimental groups after intradermal injection of HA (Profhilo) or saline solution. Hematoxylin and eosin staining (Figure 7A, upper columns) reveals the epidermis as a deep purple superficial band, with the dermis beneath it. Masson’s trichrome staining (Figure 7A, lower columns) highlights collagen fiber deposition in intense blue, mainly localized to the dermis and epidermis.

Histological sections stained with Giemsa (Figure 7B) allow visualization of mast cells in the dorsal skin. These cells appear violet in color (left and center columns), and degranulating mast cells were observed in all experimental groups (right column).

### 3.3. Changes in the Plantar Test and Spinal Cord Histology in Animals Treated Cutaneously with Saline or Ovalbumin Solution After Intradermal Injection of Hyaluronic Acid or Saline Solution

**Figure 8:**
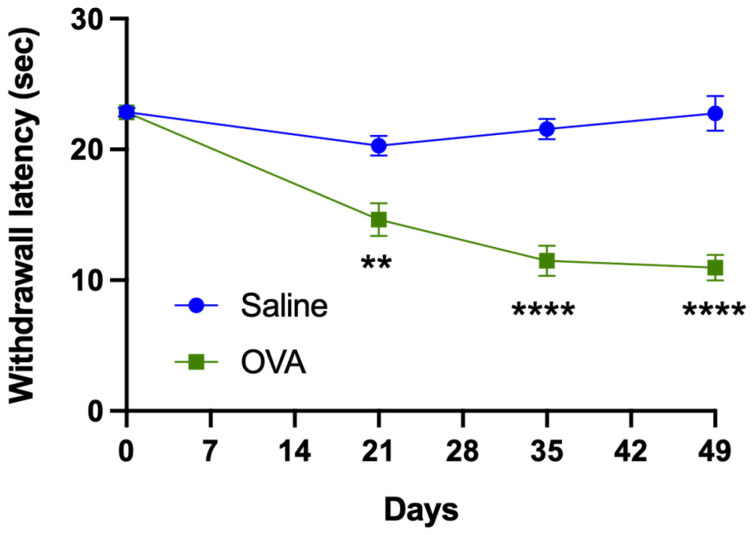
Withdrawal latency in the plantar test in animals treated cutaneously with saline solution (saline group) or ovalbumin solution (OVA group) on days 0, 21, 35, and 49 of follow-up. Animals treated with ovalbumin (OVA) show a significantly lower withdrawal latency than that observed in animals treated with saline solution on different days of follow-up, which suggests that they present thermal hyperalgesia. Values are expressed as mean ± SEM. Each experimental group included 14 animals (n = 14). *p* < 0.01 and *p* < 0.0001 compared to the control group (saline). ** *p* < 0.01; **** *p* < 0.0001.

During the first 49 days of follow-up, animals treated cutaneously with ovalbumin solution exhibited significantly shorter withdrawal latencies in the plantar thermal algesimetry test (plantar test) compared to those treated with saline (Figure 8). These findings suggest that ovalbumin treatment induces plantar thermal hyperalgesia.

On day 0, withdrawal latency in the plantar test was 22.83 ± 0.30 s in the OVA group and 22.86 ± 0.34 s in the saline group. No significant differences were observed between the two experimental groups (*p* > 0.05).

Temporal analysis of withdrawal latency in the saline group showed a significant difference only between day 0 and day 21 (*p* < 0.01). In contrast, the OVA group showed significant differences between day 0 and days 21, 35, and 49 (*p* < 0.0001), as well as between day 21 and days 35 and 49 (*p* < 0.05). No significant differences were found between days 35 and 49 (*p* > 0.05).

**Figure 9:**
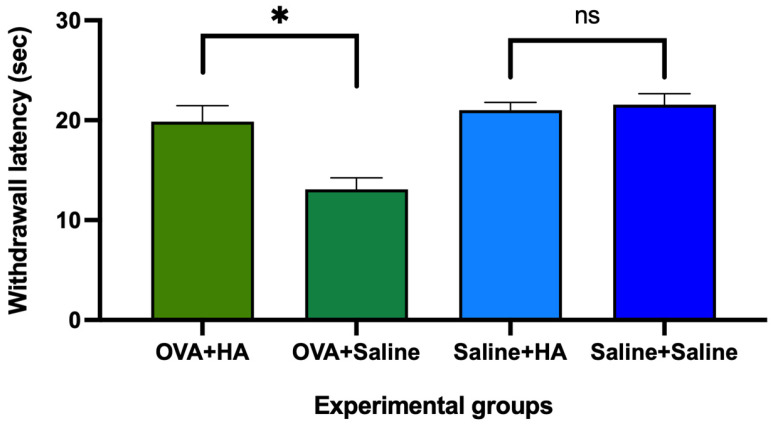
Withdrawal latency in the plantar test on day 57 of follow-up after intradermal injection of HA or saline solution. The histograms show the mean withdrawal latency and their respective mean errors in animals treated with ovalbumin (OVA) and saline solution, which were subsequently injected with hyaluronic acid (HA) or saline solution. Significant differences were observed between mice injected with HA and saline in the OVA-treated animals, but no significant differences (ns) were observed between animals injected with HA and saline that were treated with saline solution. Values are expressed as mean ± SEM. Group sizes: OVA + HA (n = 7), OVA + saline (n = 7), saline + HA (n = 8), and saline + saline (n = 6); ns = not significant. * *p* < 0.05 compared to the OVA + saline group.

On day 57 of follow-up, intradermal injection of HA significantly increased (*p* < 0.05) withdrawal latency in the plantar test in animals previously treated cutaneously with ovalbumin solution compared to animals that received intradermal saline injection. However, in animals treated cutaneously with saline solution, there were no significant differences (*p* > 0.05) in withdrawal latency between those injected intradermally with hyaluronic acid or saline solution (Figure 9).

A significant difference in withdrawal latency (*p* < 0.01) was also observed between the saline + saline and OVA + saline groups. Specifically on day 57, withdrawal latency was significantly lower in the OVA-treated group compared to the saline-treated group when both were injected intradermally with saline solution. In contrast, no significant differences (*p* > 0.05) were observed between the saline + HA and OVA + HA groups.

**Figure 10:**
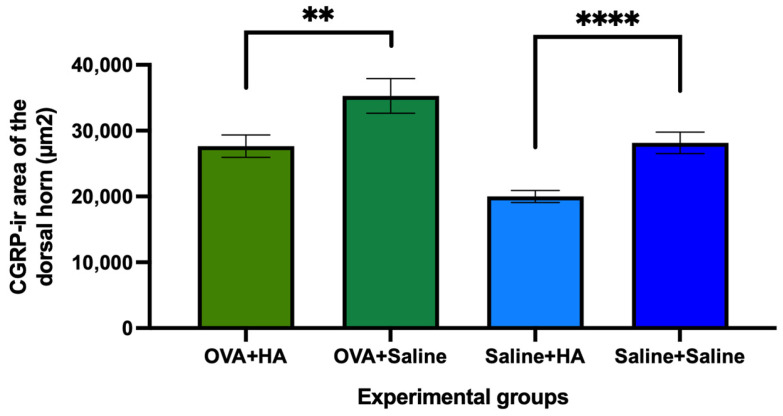
CGRP immunoreactivity (CGRP-ir) area in dorsal horn on day 57 of follow-up after intradermal injection of HA or saline solution. The histograms show the mean area of CGRP immunoreactivity in the dorsal horn and their respective errors of the mean in animals treated with ovalbumin (OVA) and saline solution, which were subsequently injected with hyaluronic acid (HA) or saline solution. Significant differences were observed between mice injected with HA and those injected with saline solution in both OVA-treated animals and saline solution. Values are expressed as mean ± SEM. Group sizes: OVA + HA (n = 7), OVA + saline (n = 7), saline + HA (n = 8), and saline + saline (n = 6). *p* < 0.01 vs. the OVA + saline group, *p* < 0.0001 vs. the saline + saline group. ** *p* < 0.01; **** *p* < 0.0001.

**Figure 11:**
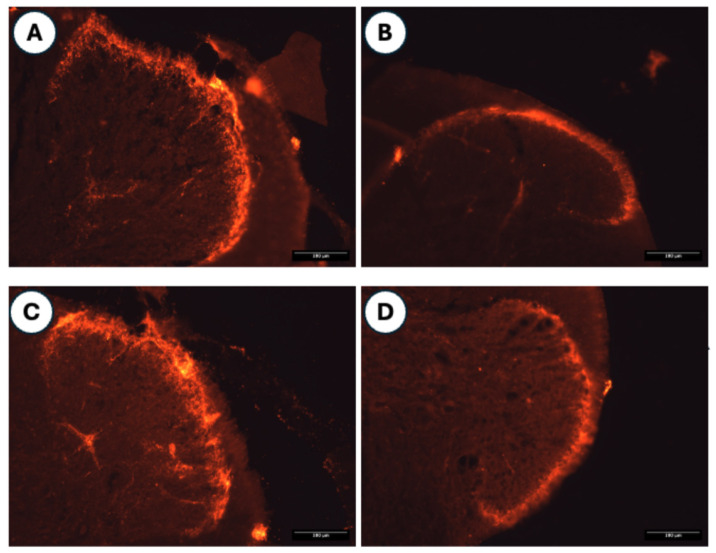
Histological images of the dorsal horn labeled with CGRP from the saline + saline (**A**), saline + HA (**B**), OVA + saline (**C**), and OVA + HA (**D**) experimental groups. The images show a histological section of the spinal cord where CGRP-positive nerve fibers appear immunolabeled in the dorsal horn. Group sizes: OVA + HA (n = 7), OVA + saline (n = 7), saline + HA (n = 8), and saline + saline (n = 6). All scale bars represent 100 µm.

Regarding the CGRP immunoreactive area (µm^2^) in the dorsal horn of the spinal cord, intradermal injection of HA resulted in a significant reduction compared to saline-injected animals, both in mice treated cutaneously with saline solution and those treated with ovalbumin solution (Figure 10 and Figure 11).

**Figure 12:**
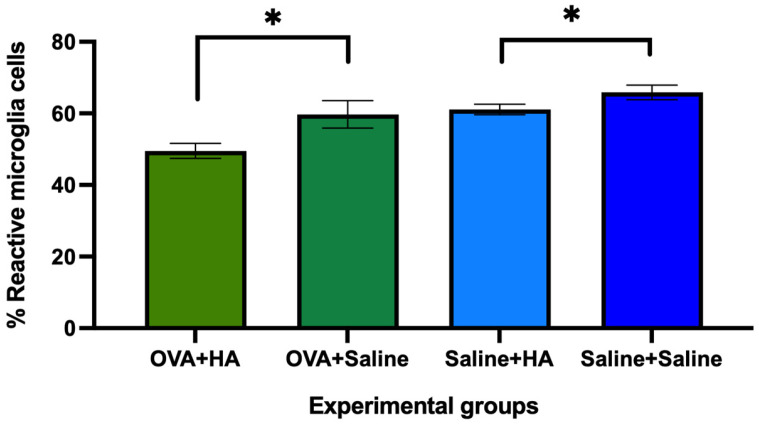
Percentage of reactive microglial cells in the spinal cord on day 57 of follow-up across the different experimental groups. The histograms show the mean percentage of reactive microglia cells and their corresponding errors of the mean in the spinal cord of animals treated with ovalbumin (OVA) and saline solution, which were also injected with hyaluronic acid (HA) or saline solution. In mice treated with OVA and saline solution, significant differences were found between mice that received HA injection compared to those that received saline injection. Values are expressed as mean ± SEM. The number of animals per group was OVA + HA (n = 7), OVA + saline (n = 7), saline + HA (n = 8), and saline + saline (n = 6). *p* < 0.05 compared to OVA + saline and saline + saline groups. * *p* < 0.05.

The percentage of reactive microglial cells in the spinal cord was significantly lower (*p* < 0.05) in animals treated with either ovalbumin or saline solution and injected intradermally with HA, compared to those injected with saline (Figure 12). No significant differences (*p* > 0.05) were observed between the saline + saline and OVA + saline groups regarding the percentage of reactive microglial cells. However, a highly significant difference (*p* < 0.0001) was found between the saline + HA and OVA + HA groups for this parameter.

These results suggest that the reduction in reactive microglia is more pronounced following hyaluronic acid injection in animals treated with ovalbumin than in those treated with saline. Nonetheless, even in saline-treated animals, intradermal injection of HA significantly reduces microgliosis.

**Figure 13:**
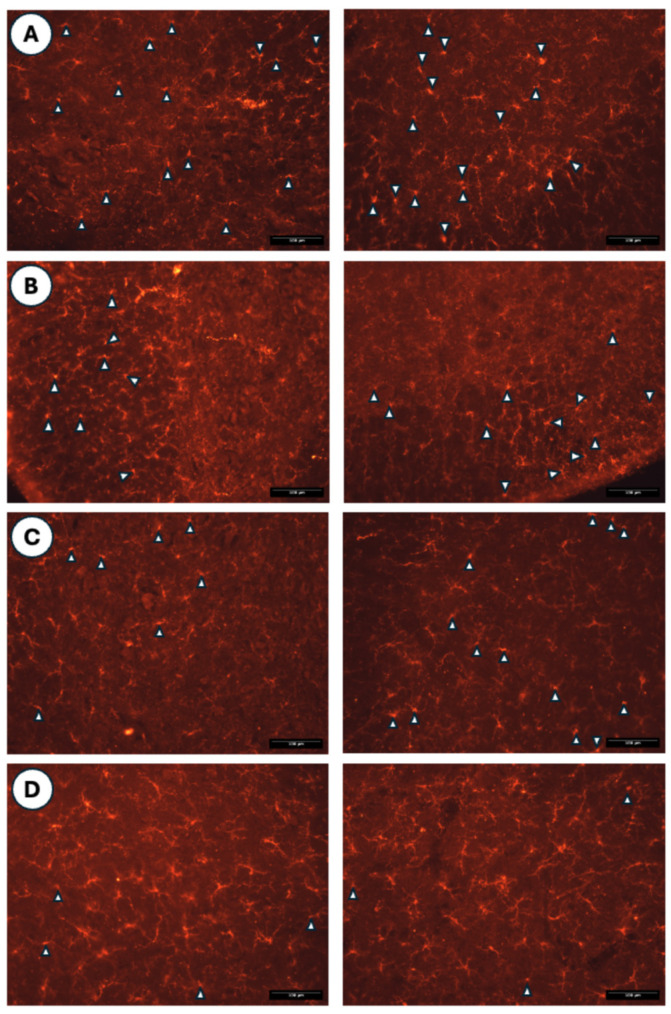
Histological images of the spinal cord immunostained for Iba1 in the saline + saline (**A**), saline + HA (**B**), OVA + saline (**C**), and OVA + HA (**D**) experimental groups. White arrowheads indicate reactive microglia, characterized by an amoeboid morphology or short cytoplasmic processes. The remaining Iba1-positive cells with long and abundant cytoplasmic processes correspond to non-reactive microglia. All scale bars represent 100 µm.

Histological sections of the spinal cord immunostained with an antibody against Iba1 are shown in Figure 13.

Table 1 shows the body weight (g) of mice treated with saline or ovalbumin solution on days 0, 21, 35, and 49 of follow-up. Table 2 presents the body weight of mice in the different experimental groups on day 57 of follow-up, after intradermal injection of HA or saline solution.

Throughout the first 49 days of follow-up, the animals’ body weight showed a slight upward trend, with no significant differences observed between the saline-treated group (saline group) and the ovalbumin-treated group (OVA group) at any time point (Table 1). On day 57 of follow-up, after intradermal injection of either HA or saline solution, no significant differences in body weight were observed between animals treated cutaneously with ovalbumin and those receiving intradermal injections of HA or saline. A similar pattern was observed in the saline-treated animals, regardless of the intradermal injection received (Table 2).

No significant differences (*p* > 0.05) in body weight were found between days 49 and 57 in any group. These results suggest that neither cutaneous administration of ovalbumin solution nor intradermal injection of HA leads to body weight loss in mice, as body weight remained slightly higher than baseline (day 0) across all groups.

Regarding the results obtained from the animal monitoring protocol, it should be noted that only the animals treated with ovalbumin showed a slight decrease in body weight during the first 21 days, which never exceeded 10%, so the score for this parameter was 1 point. Subsequently, all animals regained body weight. Furthermore, most animals appeared normal, but some mice presented skin alterations as indicated by the SCORAD score results; therefore, they received 1 point for the animal appearance parameter in the monitoring protocol. Some of these animals with skin alterations exhibited a hunched posture with closed or half-closed eyes; therefore, they received 1 point in the unprovoked behavior section. Finally, all animals in the study exhibited normal behavior in response to handling. There were no vocalizations, agitation, or aggression. Most of the animals in the study did not reach 4 points in the monitoring protocol, although it should be noted that only 3 animals had a score of 5 points, and they received treatment with glucose saline solution.

## 4. Discussion

In female ICR-CD1 mice treated cutaneously with ovalbumin solution via subcutaneous injection and topical impregnation using a brush, intradermal injection of HA (Profhilo^®^, IBSA, Italy) into the dorsal skin resulted in a significantly lower SCORAD score compared to mice treated with ovalbumin and receiving an intradermal injection of saline solution.

In addition, a significant increase in dermal thickness, but not epidermal thickness, was observed in the group treated with ovalbumin and HA compared to the saline-injected group. A decrease in mast cell density in the dorsal skin was also found in animals treated with HA relative to those receiving saline.

Furthermore, withdrawal latency in the plantar test was significantly higher in the ovalbumin-treated group that received HA compared to the group that received saline. Consistent with this, CGRP immunoreactivity in the dorsal horn of the spinal cord was significantly lower in animals treated with hyaluronic acid. Similarly, the percentage of reactive microglial cells in the spinal cord was significantly reduced in the HA group compared to the saline group.

Taken together, these findings suggest that intradermal injection of HA significantly improves dorsal skin alterations (as measured by the SCORAD scale), reduces dorsal skin mast cell density, and increases the thickness of the dorsal skin dermis. In the spinal cord subjacent to the dorsal skin, it reduces microgliosis and the sprouting of peptidergic nociceptive afferent fibers in the dorsal horn of the spinal cord. These effects were observed in an experimental model exhibiting signs consistent with atopic dermatitis.

To our knowledge, this is the first study demonstrating an effect of Profhilo^®^ HA (IBSA, Italy) in improving the signs and symptoms of a skin disorder such as atopic dermatitis. It has recently been reported that subcutaneous injection and brush application of ovalbumin–aluminum salt solution induces dermatitis-like changes in mice. Specifically, an increase in mast cell numbers, collagen deposition as revealed by Masson’s trichrome staining, and higher SCORAD scores were observed in the dorsal skin of animals treated with ovalbumin compared to those treated with saline solution [72].

In the present study, a significantly higher SCORAD score was also observed in animals treated cutaneously with ovalbumin compared to those treated with saline (see Figure 1). Erythema, edema, and dryness of the dorsal skin were the main clinical signs in animals treated cutaneously with ovalbumin solution and aluminum salts, with severities ranging from mild to moderate. Histopathological analysis suggests that the increased mast cell density in the dorsal skin may contribute to the development of these cutaneous signs. Previous studies have likewise reported erythema, edema, and dryness of the skin in ovalbumin-induced dermatitis models [70,71,72], as well as increased mast cell numbers in the skin of animals subjected to this experimental model of atopic dermatitis [66,67,72,81,82].

Preclinical evidence indicates that mast cell degranulation can be promoted by various routes of ovalbumin exposure, including intraperitoneal injection [83], oral administration [84], and inhalation [65,85].

Ovalbumin administration induces the production of IgE and IgG antibodies [86,87,88], which can bind to high-affinity IgE receptors (FcεRI) and low-affinity IgG receptors (FcγRs), respectively. Activation of these receptors initiates distinct intracellular signaling cascades within mast cells (e.g., ITAM-SfK-Syk for FcεRI and ITAM-Lyn-Syk for FcγRs), leading to the generation of second messengers such as IP3 and DAG. These, in turn, activate PKC, promoting calcium release from the endoplasmic reticulum into the cytosol. This cytosolic calcium increase is detected by the sensor proteins STIM1/2, which interact with the plasma membrane Ca2+ channel Orai-1 (calcium release-activated channel (CRAC)), resulting in influx into the mast cell. This calcium influx facilitates granule membrane fusion via activation of the SNAP-23/SNARE/STX3 complex [89,90,91,92]; this triggers mast cell degranulation, leading to the release of granule contents.

Mast cell granules contain a variety of chemical mediators, including (i) biogenic amines (e.g., histamine and serotonin), (ii) cytokines (e.g., IL-1, IL-3, IL-4, IL-5, IL-6, IL-8, IL-9, IL-10, IL-13, and IL-16), (iii) chemokines (e.g., MCP-1, MCP-3, MCP-4, MIP-1, RANTES, CXCL8, CCL2, and CCL5), (iv) polypeptides such as CRH, Endorphins, Endothelin, Kinins (bradykinin), Somatostatin, Substance P (SP), Urocortin (Ucn), VEGF, and Vasoactive intestinal peptide (VIP), (v) proteoglycans (e.g., Chondroitin sulfate, Heparin, and Hyaluronic acid), (vi) enzymes (e.g., Arylsulfatases, Carboxypeptidase A, Pro-caspase 3, 4, Chymase, b-Hexosaminidase, Kinogenases, Metalloproteinases, Nitric oxide synthase, Peroxidases, Phospholipases, and Tryptase), (vii) growth factors (e.g., SCF, GM-CSF, GnRH-I, b-FGF, NGF, and VEGF), (viii) phospholipid metabolites (e.g., LTB4, LTC4, PAF, and PGD2), and (ix) nitric oxide (NO) [93,94,95,96].

Some of these mediators promote cutaneous vasodilation (e.g., histamine, CRH, bradykinin, urocortin, VEGF, VIP, and NO), increasing blood flow to the skin and leading to redness, local heat, and edema [93]. Other mediators such as cytokines (e.g., IL-4 and IL-13), chemokines (e.g., CXCL8), and growth factors (e.g., NGF and VEGF) contribute to epidermal barrier disruption and increased skin permeability [97], promoting cutaneous water loss and resulting in skin dryness.

Skin itching and scratching can further compromise the epidermal barrier, exacerbating skin dryness. Additionally, certain chemical mediators released during mast cell degranulation (e.g., histamine, serotonin, and bradykinin) interact with their respective membrane receptors (e.g., H1, 5-HT3, and B1) on nociceptive/pruritogenic nerve fibers in the skin, causing depolarization of these fibers [98,99,100], which leads to the sensation of itch and activation of the scratch reflex [101]. Scratching and skin friction also promote redness and increase transepidermal water loss, further contributing to skin dryness [102].

Chemical mediators released by mast cells during degranulation also promote peripheral sensitization of these fibers (e.g., bradykinin, histamine, and prostaglandins) and their hyperexcitability (e.g., prostaglandins) by enhancing ionic currents through voltage-gated sodium channels [103,104,105]. The excitability, hyperexcitability and sensitization of these afferent nociceptive/pruritogenic fibers lead to the release of neurotransmitters (e.g., glutamate, CGRP, SP, and ATP) over second-order nociceptive/pruritogenic neurons in the dorsal horn of the spinal cord, thereby transmitting sensory signals via the ascending pathways of the spinal cord [106,107,108]. These neurotransmitters also interact with receptors located on microglia cells in the dorsal horn of the spinal cord [79,109], triggering the reactivation of these glial cells, which in turn release cathepsin-S, which facilitates the generation of fractalkine and promotes the synthesis of pro-inflammatory mediators (e.g., IL-1, TNF-alpha, and IL-6) that contribute to the excitation and sensitization of second-order nociceptive neurons in the dorsal horn of the spinal cord (a phenomenon known as central sensitization) [110]. Central sensitization of spinal nociceptive neurons driven by chemical mediators released from reactive microglia contributes to enhanced pain sensitivity, or hyperalgesia [111,112].

These mechanisms may help to explain the plantar thermal hyperalgesia observed in animals treated with ovalbumin compared to those treated with saline, which also show a higher density of mast cells (see Figure 7 and Figure 9). Furthermore, reactive microglial cells were observed in histological sections of the spinal cord from both ovalbumin- and saline-treated animals (see Figure 13), although no significant differences were noted between animals injected intradermally with saline.

The novelty of the present study lies in the finding that intradermal injection of HA in animals treated with ovalbumin significantly (i) reduces the SCORAD score, (ii) relieves plantar thermal hyperalgesia, (iii) reduces the density of mast cells in the dorsal skin, (iv) increases the dermal thickness of the dorsal skin, (v) reduces the percentage of reactive microglial cells in the spinal cord, and (vi) decreases the area of CGRP-immunoreactive fibers in the dorsal horn.

Clinical studies show that topical HA treatment reduces erythema and pruritus in seborrheic dermatitis [4], decreases trans-epidermal water loss and erythema, and relieves pruritus [52], and improves the SCORAD score [113] in subjects with atopic dermatitis. All this evidence suggests that treatment with HA reduces erythema and pruritus, two key parameters contributing to improvements in the SCORAD score.

HA is known to bind to CD44 receptors on mast cells [114], and this inhibits the activation of protein kinase C (PKC) [115], which prevents the mobilization of calcium in the mast cell cytoplasm, and this contributes to reducing mast cell degranulation; therefore, HA decreases mast cell degranulation [115]. By limiting the release of mast-cell-derived chemical mediators that promote vasodilation, erythema, and edema, HA may help improve the SCORAD score in conditions involving cutaneous inflammation, such as atopic dermatitis. This effect was observed in the present study following intradermal injection of hyaluronic acid and has also been reported by other researchers [9,113,116].

Several experimental studies of dermatitis demonstrate that treatments reducing the number of mast cells in the skin and their activation are associated with improvements in the SCORAD score [10,117,118,119,120]. The presents study also observed that a significant decrease in mast cell numbers in the dorsal skin, following intradermal injection of HA in animals with ovalbumin-induced AD-like symptoms, was accompanied by a significant improvement in the SCORAD score.

The dermal thickness observed in animals with ovalbumin-induced atopic dermatitis that received intradermal injections of HA can be partly explained by the fact that HA promotes fibroblast proliferation [121,122] and that intradermal injection of cross-linked HA (Restylane) promotes the synthesis, release, and deposition of type 1 collagen in the dermis [123], as well as the fact that dermal injection of HA increases dermal thickness [124,125].

HA also influences the biology and physiology of epidermal keratinocytes, potentially affecting epidermal thickness. In keratinocytes, hyaluronic acid binds to CD44 receptors on the cell membrane of keratinocytes, activating two distinct intracellular signaling pathways: a RhoA-ROK-dependent pathway, which promotes cell growth and survival, and a Rac1-PKN-dependent pathway, which stimulates keratinocyte differentiation and cell-cell adhesion [126].

However, the behavior of epidermal keratinocytes is also regulated by soluble factors released by dermal fibroblasts, which are themselves stimulated by HA. Hyaluronic acid promotes the synthesis and release of cytokines (IL-1β, TNF-α, and IL-8), growth factors (VEGF, FGF2, b-FGF, and TGF-β1) [127,128,129], and overexpression of COX2, which in turn increases the synthesis and release of prostaglandins (PGE2) [130]. These chemical mediators released by dermal fibroblasts in response to HA promote keratinocyte migration, growth, and proliferation [131,132,133,134]. However, other chemical mediators (TGF-β1 and TNF-a) inhibit proliferation/growth [135,136] and promote apoptosis [137,138] of keratinocytes. Overexpression of these factors has been reported after atopic dermatitis [139,140]. These findings suggest that while HA can promote keratinocyte growth and proliferation, potentially leading to increased epidermal thickness, this effect was not observed in the present study following intradermal injection of HA. The increase in fibroblast chemical messengers that inhibit proliferation and promote keratinocyte apoptosis may partially explain the absence of epidermal thickening.

The significant relief of thermal hyperalgesia observed in ovalbumin-induced AD-like mice after intradermal injection of HA compared to animals that received intradermal saline may be explained by the reduction in mast cell numbers and the decreased release of chemical mediators from these cells, as previously suggested. This leads to reduced excitability and peripheral sensitization of nociceptive and pruritogenic nerve fibers in the skin. However, HA also binds to the TRPV1 receptor of nociceptors, causing them to open less frequently, which reduces their excitability and their response to painful stimuli [141].

In an experimental model of intervertebral disc injury, application of an HA hydrogel reduced nociceptive behaviors such as plantar thermal hyperalgesia, mechanical allodynia, and the tail-flick response while also decreasing TRPV1 expression in CGRP-positive nociceptive nerve fibers [142]. Furthermore, intradermal injection of prostaglandins (PGE2) into the skin of the dorsum of the paw induces mechanical hyperalgesia, which is attenuated by injecting 5 µL of high molecular weight hyaluronic acid (HA) into the same area 10 min later. This anti-hyperalgesic effect of HA is mediated by interaction with the CD44 receptor: if a CD44 receptor antagonist is injected after PGE2 but before hyaluronic acid, the anti-hyperalgesic effect of HA is reversed [143]. On the other hand, in animals initially injected with low-molecular-weight HA (which causes mechanical hyperalgesia), a subsequent injection of high-molecular-weight HA significantly reverses the mechanical hyperalgesia induced by low-molecular-weight HA [144]. It should be noted that nociceptive C-peptidergic and pruritogenic nerve fibers express TRPV1 and CD44 receptors, to which HA binds, modulating their excitability [145,146], thereby reducing responses to pain.

This anti-hyperalgesic effect of HA applied to the skin can be translated into a reduction in microgliosis within the spinal cord. Ovalbumin applied to the skin triggers mast cell degranulation, releasing chemical mediators that excite and sensitize cutaneous nociceptors. This, in turn, causes persistent reactivation of spinal cord microglia, which secrete further chemical mediators that sensitize second-order nociceptive neurons. These chemical mediators derived from reactive microglia diffuse through the spinal cord parenchyma, causing remote reactivation of microglia cells in caudal regions of the spinal cord (lumbosacral regions), which in turn synthesize and secrete the same chemical mediators that excite and sensitize the spinal nociceptive neurons of these lumbosacral regions of the spinal cord [147,148]. This response is consistently observed in animals treated with ovalbumin compared to controls up to 49 days post-treatment. On the other hand, previous experimental evidence suggests that intradermal injection of HA alleviates mechanical hyperalgesia (measured using an analgesymeter) in animal models of chemotherapy-induced neuropathic pain [149], whereas after spinal cord injury at the thoracic level, it induces remote lumbosacral microglia cells that increase mechanical hyperalgesia and elevated levels of inflammatory mediators (IL-1, TNF-alpha, and IL-6) which are also associated with greater mechanical hyperalgesia [147]. These findings suggest that reactivation of remote microglial cells in lumbosacral regions could underlie plantar hyperalgesia, and HA can modulate microglial reactivity and hyperalgesia.

Experimental evidence suggests that NGF induces extensive branching of CGRP-positive afferent fibers in the dorsal horn [150], and that microglia reactivated by cytokines or LPS secrete NGF [151,152]. Moreover, mast cells are known to synthesize, store, and secrete NGF [153,154]. This evidence suggests that both mast cells and reactive microglial cells are sources of NGF, a neurotrophic factor that promotes sprouting of CGRP-positive afferent fibers in the spinal cord.

In experimental models of ovalbumin-induced atopic dermatitis, as in the present study, an increased number of mast cells in the dorsal skin has been observed following ovalbumin treatment [66,67,72,81,82], as well as an increase in reactive microglial cells in the spinal cord [75]. Both cell types (mast cells and microglia) are expected to secrete this growth factor (NGF) following ovalbumin administration, similar to what is observed in the experimental model of ovalbumin-induced allergic asthma, where NGF levels increase [155].

NGF binds to TrkA receptors expressed by CGRP-positive nociceptive nerve fibers, promoting the release of neurotransmitters from these peptidergic nociceptive fibers to second-order nociceptive neurons and glial cells in the dorsal horn of the spinal cord [156]. Simultaneously, it promotes the sprouting of these nerve fibers in both the skin and the dorsal horn of the spinal cord, which is associated with increased thermal sensitivity and mechanical hyperalgesia [157]. In addition, elevated levels of NGF have been observed following mast cell proliferation after allergen application [158], reactive microglia proliferation following CNS injury [159], and in subjects with atopic dermatitis [160]. NGF itself is also known to induce mast cell degranulation [161]. Taken together, these findings suggest that NGF secreted by mast cells and reactive microglia may be involved in the peripheral and central branching of peptidergic nociceptive nerve fibers, which may contribute to hyperalgesia.

In the present study, it was observed that animals treated with ovalbumin and subsequently given intradermal injections of HA showed a reduction in mast cell numbers in the dorsal skin, as well as a decreased percentage of reactive microglia in the spinal cord and a reduction in the area of CGRP immunoreactivity in the dorsal horn of the spinal cord. All of this translated into a relief of plantar thermal hyperalgesia, compared to animals that received intradermal injections of saline solution. As previously mentioned, HA has anti-hyperalgesic effects [143,149], reduces the number and proliferation of mast cells in the skin [10,117], and decreases mast cell degranulation [115]. There is also evidence that HA reduces microglial cell activation [162] and lowers NGF levels [163]. All these effects could explain the decreased area of CGRP immunoreactivity in the dorsal horn of ovalbumin-treated animals that received intradermal injections of HA.

Some limitations and future directions of this study are (i) although calculated, the number of animals per experimental group may be a limiting factor, and therefore the study could be replicated with a larger number of animals; and (ii) only female mice were used in the study, and therefore the results should be considered only for this sex. The study could be replicated in male mice to determine if there are potential sex-dependent differences; (iii) the study includes functional and histological evaluation of the observed skin changes but lacks molecular analysis. The absence of these molecular results prevents us from determining whether the treatment used induces variations in the levels of cytokines and/or chemical mediators released by mast cells/microglia, and therefore the discussion has been based on previous evidence from other scientific studies. Future studies should include molecular analysis of the skin and spinal cord; and (iv) the experimental model allows the use of different treatments already used in human subjects or new preclinical treatments that improve the signs and symptoms of atopic dermatitis.

## 5. Conclusions

This study demonstrates that intradermal injection of hyaluronic acid (Profhilo^®^), a hybrid complex of high- and low-molecular-weight HA, exerts significant therapeutic effects in a mouse model of ovalbumin-induced atopic dermatitis (AD). Treatment with HA resulted in marked reductions in skin inflammation, as evidenced by lower SCORAD scores and decreased mast cell density, along with increased dermal thickness. Additionally, HA alleviated pain-related behavior by reversing thermal hyperalgesia and modulating spinal neuroimmune responses through reduced CGRP immunoactivity and microglial activation. These findings suggest that HA not only improves local cutaneous pathology but also influences central sensitization mechanisms associated with chronic inflammation. The results support the potential of intradermal HA formulations, such as Profhilo^®^, as novel therapeutic agents for managing AD and related neuro-immune skin disorders.

## Figures and Tables

**Table 1:** Body weight (g) of mice in the two experimental groups during follow-up (days 0 to 49). Values are expressed as mean ± SEM.

Day	Saline Group	OVA Group	
0	33.67 ± 0.67	33.68 ± 0.53	*p* = 0.6703
21	34.13 ± 0.68	33.00 ± 0.90	*p* = 0.3635
35	34.67 ± 0.71	34.08 ± 0.83	*p* = 0.5265
49	39.27 ± 1.17	36.54 ± 0.76	*p* = 0.0967

**Table 2:** Body weight (g) of mice in the different experimental groups on day 57 of follow-up. Values are expressed as mean ± SEM.

Experimental Group	Body Weight (g)	
OVA + HA	36.14 ± 1.60	*p* = 0.9818
OVA + Saline	35.75 ± 1.44	
Saline + HA	36.88 ± 1.60	*p* = 0.1417
Saline + Saline	41.86 ± 2.04	

## Data Availability

All data generated or analyzed during this study are included in this published article.

## Abbreviations

The following abbreviations are used in this manuscript:

AD	Atopic dermatitis
ATP	Adenosine triphosphate
B1	Bradykinin receptor type 1
BDDE	1,4-butanediol di-glycidyl ether
BIS	(-)-α-Bisabolol
CCL2	Chemokine (C-C motif) ligand 2
CCL5	Chemokine (C-C motif) ligand 5
CGRP	Calcitonin gene-related peptide
CNS	Central nervous system
COX2	Cyclooxygenase 2
CRH	Corticotropin-releasing hormone
CXCL8	C-X-C motif chemokine ligand 8
Cy3	Cyanine 3.18
DAG	Diacylglycerol
DPX	Dibutylphthalate polystyrene xylene
DNCB	2,4-dinitrochlorobenzene
DNFB	2,4-dinitrofluorobenzene
FcεRI	High-affinity IgE receptors
FDA	Food and drug administration
FcγRs	Low-affinity IgG receptors
b-FGF	Basic fibroblast growth factor
FGF2	Fibroblast growth factor 2
GM-CSF	Granulocyte-macrophage colony-stimulating factor
GnRH-I	Gonadotropin-releasing hormone I
H1	Histamine receptor type 1
5-HT3	Serotonin receptor type 3
HA	Hyaluronic acid
HDM	House dust mites
Iba1	Ionized calcium-binding adapter molecule type 1
IBSA	Institut biochimique SA
ICR-CD1	Institute of cancer research (ICR)-CD1 mice
IgE	Immunoglobulin E
IL-1	Interleukin 1
IL-3	Interleukin 3
IL-4	Interleukin 4
IL-5	Interleukin 5
IL-6	Interleukin 6
IL-8	Interleukin 8
IL-9	Interleukin 9
IL-10	Interleukin 10
IL-13	Interleukin 13
IL-16	Interleukin 16
IP3	Inositol triphosphate
LPS	Lipopolysaccharide
LTB4	Leukotriene B4
LTC4	Leukotriene C4
MCP-1	Monocyte chemotactic protein 1
MCP-3	Monocyte chemotactic protein 3
MCP-4	Monocyte chemotactic protein 4
MC903	Calcipotriol
MIP-1	Macrophage inflammatory protein 1
NAHYCO	Nahyco^®^hybrid technology
NGF	Nerve growth factor
NIH	National institutes of Hhalth
NO	Nitric oxide
OVA	Ovalbumin
OVA-AL	Ovalbumin aluminum salt
PAF	Platelet-activating factor
PBS	Phosphate-buffered saline
PGD2	Prostaglandin D2
PGE2	Prostaglandin E2
PKC	Protein kinase C
Rac1	Rac family small GTPase 1
RANTES	Regulated on activation, normal T-cell expressed and secreted
RhoA	Ras homolog family member A
ROK	Rho kinase
SADBE	Squaric acid dibytylester
SCORAD	SCORing atopic dermatitis
SCF	Stem cell factor
SEM	Standard error of the mean
SNAP-23	Synaptosomal-associated protein 23
SNARE	Soluble NSF attachment protein receptor
STIM1/2	Stromal interaction molecule 1 and 2
STX3	Syntaxin 3
SP	Substance P
TGF-β1	Transforming growth factor-β1
TNF-alpha	Tumor necrosis factor alpha
TPA	12-O-tetradecanoylphorbol-13-acetate
TRPV1	Transient receptor potential vanilloid subtype 1
Ucn	Urocortin
VEGF	Vascular endothelial growth factor
VIP	Vasoactive intestinal peptide
W	Watts
